# Effect of finasteride particle size reduction on its pharmacokinetic, tissue distribution and cellular permeation

**DOI:** 10.1080/10717544.2018.1440446

**Published:** 2018-02-16

**Authors:** Tarek A. Ahmed, Ahmed M. Al-Abd

**Affiliations:** a Department of Pharmaceutics, King Abdulaziz University, Jeddah, Kingdom of Saudi Arabia;; b Department of Pharmaceutics and Industrial Pharmacy, Al-Azhar University, Cairo, Egypt;; c Department of Pharmacology, King Abdulaziz University, Jeddah, Kingdom of Saudi Arabia;; d Department of Pharmacology, Medical Division, National Research Centre, Giza, Egypt

**Keywords:** Finasteride, micro and nanoparticles, pharmacokinetics, tissue distribution, BPH

## Abstract

Finasteride (FSD), a specific competitive inhibitor of the steroid type-II 5α-reductase enzyme, is used in treatment of benign prostate hyperplasia (BPH) and male pattern baldness. The drug is of limited solubility that affect its dissolution and bioavailability. The aim was to study the effect of FSD particle size reduction on the pharmacokinetic, tissue distribution and cellular permeation. An optimized drug micro- and nano-particles were developed, characterized, administered to group of rats, and systemic pharmacokinetic and tissue distribution within target and not-target organs were determined using near-infrared (NIR) spectroscopy technique. Moreover, the cellular permeation of the prepared formulations through normal prostate epithelial cells was assessed and compared to pure FSD. The developed micro- and nano-particles were of 930 and 645 nm, respectively. Plasma maximum drug levels (C_max_) and overall exposure (AUC) of both formulations were not significantly higher than unformulated drug. However, micronized FSD achieved significant higher concentration within the target tissue (prostate) within the current study compared to pure drug and nano-sized formulation as well. Yet, this is explained by the higher sequestration ability of spleen tissue to the nano-sized formula compared to micro-sized FSD. At the cellular level, permeation of nano-sized FSD through prostate epithelial cells was superior to the unformulated FSD as well as the micro-sized drug formulation. FSD particle size reduction significantly influences its cellular permeation and to a lesser extend affect its systemic pharmacokinetics and tissue distribution after oral administration.

## Introduction

Size reduction is a process of decreasing the large or coarse solid drug particles into small or fine unit masses. This process represents a potentially useful way for enhancing the bioavailability of hydrophobic drugs especially when particles in the nano-size range have been developed (Wang et al., 2013). Improving drug bioavailability due to size reduction is attributed to the increase in the drug surface area, solubility and dissolution rate (Jinno et al., 2006; Junghanns & Müller, 2008; Willmann et al., 2010). Spray and freeze drying, anti-solvent precipitation, crystallization, supercritical fluid technology, high pressure homogenization, ball milling, jet milling, crystal engineering and cryogenic spray process are techniques commonly employed to achieve size reduction (Rogers et al., 2001; Blagden et al., 2007; Sutradhar et al., 2013; Wang et al., 2013; Hao et al., 2015). The bioavailability and therapeutic activity of wide variety of drugs have been successfully augmented following size reduction of these drug particles (Van Eerdenbrugh et al., 2008; Pande & Abhale, 2013; Wang et al., 2013).

Drug pharmacokinetic inspects the change in body drug concentrations with respect to time as a function of absorption and disposition. The later represents a combination of distribution and elimination (Maureen Dale & Rang, 2012). Following absorption, the administered drug is distributed reversibly to different body tissues and organs including those responsible for the elimination, such as the liver and the kidney, which results in a marked decrease in blood drug concentration. For poorly soluble drugs that are administered orally, there is always a low concentration gradient between the amount of drug at the absorption site and blood vessels which leading to inadequate drug absorption and distribution. Increasing both the drug solubility and dissolution rate via particle size reduction is considered as a promising approach in improving the drug pharmacokinetic and therapeutic activity (Sun et al., 2012; Hao et al., 2015; Rodrigues et al., 2015).

Finasteride (FSD), 17β-(N-tert-butylcarbamoyl)-4-aza-5α-androst-1-*en*-3-one, is an orally active inhibitor of the intracellular enzyme 3-oxo-5-α-steroid 4-dehydrogenase that is responsible for conversion of androgen testosterone into 5α-dihydrotestosterone (Tian et al., 1994). A decrease in the blood level of the later will result in a decrease in prostatic volume. FSD is considered a surgical alternative oral medication used in the treatment of benign prostatic hyperplasia and in the treatment of mild to moderate androgenetic alopecia (Prahalada et al., 1994). The drug characterized by its limited aqueous solubility that affects both; dissolution and bioavailability. FSD size reduction could be considered a promising strategy in enhancing the solubility and accordingly the dissolution rate and drug bioavailability. Different formulations have been reported to enhance FSD bioavailability, through oral and non-oral routes, especially when the drug is formulated in a nanostructured systems (Monti et al., 2014; Tampucci et al., 2014). Also, drug disposition and intracellular activity on BPH cells could be improved when employing the same technique.

This study represents the second step in the implementation of size reduction technique in achievement of enhanced drug tissue distribution and cellular permeation. An optimized drug micro and nanoparticles were prepared, characterized and their tissue distribution in the body vital organs and permeation through BPH cells were estimated. Also, near-infrared (NIR) spectroscopy has been employed to assess the drug level in the studied organs.

## Experimental methods

### Materials

FSD was obtained as a kind gift from Saudi Arabian Japanese (SAJA) Pharmaceutical Company Limited (Jeddah, Saudi Arabia). Polyvinyl alcohol (PVA) was purchased from Spectrum Chemicals & Laboratory Products (Gardena, CA). Acetonitrile of high-performance liquid chromatography (HPLC) grade was procured from Fisher Scientific UK (Loughborough, Leicestershire, UK). Methanol was purchased from Sigma-Aldrich (St. Louis, MO).

### Methodology

#### Preparation of FSD micro and nanoparticles

An optimized drug micro and nanoparticles were prepared according to our previously published work (Ahmed, 2016; Ahmed & El-Say, 2016). The condition utilized to prepare both formulations is summarized in Table 1. For preparation of FSD microparticles (m-FSD); an alcoholic drug solution (100 mg/mL) was prepared by dissolving the drug in methanol then, was slowly injected into 50 mL of 0.2% w/v PVA solution, and the obtained dispersion was kept on a magnetic stirrer at 1000 rpm until complete evaporation of methanol. The volume ratio of the methanolic drug solution to the aqueous PVA solution was kept at 1:10. The obtained drug suspension was ultrasonicated for 10 min under ice cooling condition using Sonics VCX 750, Sonics & Materials Inc. (Newtown, CT). Dried m-FSD were obtained after freeze drying for 48 h at −90 °C under 0.01 mbar pressure using alpha 1–2 LD plus, Christ lyophilizer (Osterode am Harz, Göttingen, Germany). For preparation of FSD nanoparticles (n-FSD); an alcoholic drug solution (100 mg/mL) was prepared and added dropwise into the same volume of 0.67% w/v PVA aqueous solution on a magnetic stirrer at 1000 rpm at a ratio of 0.154:10 alcoholic to PVA aqueous phase. The mixture was left on a magnetic stirrer at 1000 rpm until evaporation of methanol was completed. The aqueous drug dispersion obtained was homogenized at 17,660 rpm using UltraTurax, IKA^®^ T18 basic Homogenizer (IKA, Campinas, Brazil) for 10.584 min. Dried n-FSD were obtained following freeze drying as previously described.

**Table 1.** Optimum preparation condition and characteristics of FSD micro and nanoparticles.

	Optimum preparation condition				Particles characteristics		
Formulation	X1	X2	X3	X4	Size (nm)	ZP (mv)	SE (%)
m-FSD	0.2	10	–	–	930	2.40	124
n-FSD	0.669	1.542	17,660	10.584	645	2.42	159.24

X1: PVA concentration; X2: ratio of methanolic solution; X3: homogenization speed (rpm); X4: time of homogenization (min); ZP: zeta potential; SE: solubility enhancement.

#### Characterization of the prepared micro and nanoparticles

The obtained m-FSD and n-FSD were subjected to particle size and zeta potential measurement using Zetatrac of Microtrac Inc. (Montgomeryville, PA). Solubility enhancement of the developed particles was assessed after evaluation the solubility of the pure drug and the solubility of the prepared m-FSD and n-FSD in distilled water. Briefly, excess amount of each sample was placed in a screw cap glass vial containing 10 mL of distilled water and the vials were kept shaking at room temperature for 72 h in a thermostatically controlled shaking water bath (Model 1031; GFL Corporation, Burgwedel, Germany). Aliquots of each vial were withdrawn, filtered using Acrodisc^®^ syringe filter of 0.45 µm, and analyzed for the drug concentration in the clear filtrate using HPLC (Ahmed & El-Say, 2016). The solubility enhancement was estimated using the following equation:

$$SE\;=\frac{A-B}{A}\times 100$$

where SE is the solubility enhancement, A is the aqueous solubility of the prepared m-FSD or n-FSD formulations and B is the aqueous solubility of the pure FSD.

*In vitro* dissolution of the pure FSD powder and the prepared m-FSD and n-FSD was performed using United States Pharmacopeia (USP) dissolution test apparatus II paddle type, DT 700 LH device, Erweka GmbH DT 700 (Heusenstamm, Germany) at 37 °C for 2 h, with a stirring rate of 50 rpm, in 500 mL distilled water containing 0.05% sodium lauryl sulfate, to provide sink condition. Samples (*n* = 3) were withdrawn and analyzed for drug content using HPLC.

#### Construction of FSD calibration curve in plasma and tissue homogenate

Plasma and tissue homogenates samples spiked with FSD were prepared by adding known concentration of the drug to 2 mL of either blank plasma or tissue homogenates to reach a concentration range of 5–200 ng/mL for calibration analysis. The prepared samples were frozen at −80 °C and then subjected to freeze drying using alpha 1–2 LD plus, Christ lyophilizer.

The prepared plasma and tissue homogenates samples were subjected to NIR analysis using Thermo Fisher Scientific NICOLET iZ 10, Integrating Sphere Near-IR Spectrometer (Madison, WI). The spectra for pure FSD, freeze-dried drug free plasma and freeze-dried FSD plasma samples were recorded over the wave length range 4000–10,000 cm^−1^ against % transmittance to identify the characteristic NIR drug frequency region (finger print). The studied samples were measured without any sample preparation. The transmission spectra collected for each sample were the average of 36 scans and were visualized and chemometrically processed using the TQ Analyst software (tenth edition; Thermo Fisher Scientific).

For calculation of the drug concentration, partial least square (PLS) regression was used to relate the obtained spectroscopic data to the drug concentration. PLS is a factor which process all the data from a full spectrum and then uses only the relevant factors that are present in the spectral data (Merckle & Kovar, 1998). This factor is used to develop a calibration model based on the data of the calibration set which can then be used to expect the drug concentration from the spectra of the test set.

Selectivity was tested through assessment of the potential interference at the characteristic drug frequency region with endogenous components. Linearity of the method was studied for the samples used for construction of the calibration curve in plasma and tissue homogenate in the concentration range, 5–200 ng/mL where linear regression estimated the goodness-of-fit. Intra and interday precision and accuracy were evaluated by analysis of three replicates of plasma and tissue homogenate samples containing three different concentrations on the same and three separate days. For assessment of the method precision, the relative standard deviation was calculated according to the following equation:

$$Relative\;standard\;deviation=\frac{Calculated\;standard\;deviation}{mean\;observed\;concentration}\;\times 100$$

Method accuracy was calculated as the mean concentration found relative to the actual concentration.

#### Animals

Male Sprauge Dawly rats (weight 150–175 g) were maintained in the pathogen-free area of the Pharmacology and Toxicology Department, Faculty of Pharmacy, King Abdulaziz University (Jeddah, KSA). Animals had access to food and water *ad labium*. Animal experimental protocol was approved by the Ethical and Animal Care Committee at Faculty of Pharmacy, King Abdulaziz University.

### Pharmacokinetics and tissue distribution of FSD after oral administration of different formulations

To determine the influence of different formulation on the plasma pharmacokinetics and tissue distribution of FSD, rats were orally given suspension of 0.4 mg/kg pure FSD (c-FSD), m-FSD or FSD nanoparticles (n-FSD) in CMC solution. Animals were anesthetized and blood samples were taken at different time points (0.5, 1, 1.5, 2, 4, 6, 8, 12 and 24 h). Immediately afterward, animals were euthanized by cervical dislocation, and prostate, liver, spleen, kidney, lung and testis were harvested within 20 min and stored at −80 °C to be assayed. The collected plasma samples were frozen at −80 °C and subjected to freeze drying while, the tissue samples were homogenized in PBS (20% w/v) before they were subjected to freeze drying. Assayed for FSD content was achieved using Near IR method previously described in the above section.

### Cell culture and cellular pharmacokinetics

To assess the ability of different FSD formulation to permeate cell membrane of prostate tissue, we measured the ability of FSD and different formulations to penetrate to the intracellular compartment of prostate cells. In brief, normal human prostate epithelial cells were generously gifted from Dr. Zakaria Abd Elmageed (College of Pharmacy, Texas A&M Health Science Center, Kingsville, TX), and maintained in RPMI-1640 media supplemented with 100 µg/ml streptomycin, 100 units/ml penicillin and 10% heat-inactivated fetal bovine serum in a humidified chamber at 37 °C supplied with 5% (v/v) CO_2_.

For cellular pharmacokinetics assessment, 1 × 10^6^ viable cells were seeded and left to attach for 24 h. Cells were exposed to suspension of the different formulations of FSD (10 µg/ml) and collected by trypsinization after 1, 3, 6, 24 and 48 h. Cells were washed trice with ice-cold PBS and then ruptured by sonication in hypotonic saline solution (1 ml) for 20 min. Ruptured cells were mixed with acetonitrile (1 ml), acetone (250 µl) and saturated ZnSO_4_ solution (100 µl) centrifuged at 13,200 rpm for 10 min to remove debris. FSD in the supernatant was assayed using HPLC of Agilent 1200 series equipped with UV, diode array detector. Agilent C18 100 mm ×3.3 mm, 4.5 μm particle size analytical column (Agilent Technologies, Inc., Littelfall, CA) was used. An isocratic mobile phase consists of acetonitrile:15 mM phosphate buffer:tetrahydrofuran (42:55:3) v/v was delivered at a flow rate of 0.4 ml/min and the detector wave length was set at 220 nm. The HPLC method was reproduced except for slight modification to that previously published by Anutra et al. (2003).

### Statistical analysis

Data are presented as mean ± standard error of mean (SEM). Analysis of variance (ANOVA) with LSD *post hoc* test was used for testing the significance using SPSS^®^ for windows, version 17.0.0 (Chicago, IL). *p* < .05 was taken as a cut off value for significance.

## Results and discussion

The concept of particle size reduction, which is accompanied by increase in the particle surface area and drug solvation, is well-known to increase the drug dissolution rate as previously described by the famous Noyes–Whitney equation (Dokoumetzidis & Macheras, 2006). The technique is therefore usually used to enhance the bioavailability of poorly soluble drugs (Sun et al., 2012).

Several formulation and processing factors were studied for their effect on the development of m-FSD and n-FSD. PVA was selected as a stabilizer. It is a water soluble protective colloid that is adsorbed on the particle surface and facilitate formation of small size and stable particles during the size reduction process (Ahmed, 2016). The concentration of PVA was found to be critical since, insufficient PVA concentration will not provide adequate coverage of the particles, while at higher PVA concentration polymeric micelles will be formed that affect the thermal stability of the prepared particles (Wang et al., 2013). Other studied parameters such as the ratio of methanolic solution, homogenization speed and time of homogenization were also studied, and their optimum levels are presented in Table 1. Discussion about the effect of each factor and the optimum concentration were illustrated in our previously published work (Ahmed, 2016; Ahmed & El-Say, 2016).

Results of the characteristics for the prepared m-FSD and n-FSD are illustrated in Table 1. m-FSD and n-FSD of 930 and 645 nm, respectively were obtained. Zeta potential, which reflects the particles surface charges, was ∼2.40 mV. Enhancement of the drug solubility following formulation of m-FSD and n-FSD was found to be 124 and 159.24%, respectively. *In vitro* dissolution revealed enhancement of the rate of drug dissolution from m-FSD and n-FSD by 2–2.5 times when compared with pure FSD powder (data not shown). Enhancement of the drug solubility and dissolution is directly related to size reduction, the effect which was significant in n-FSD when compared to m-FSD since the former possess smaller size than the later. Flakes like, non-spherical, short rods small plates were the morphology of the obtained microparticles as indicated by images of the scanning electron microscope (Ahmed & El-Say, 2016), while small homogenous spherical particles were noticed for the n-FSD (Ahmed 2016). Physicochemical characterization of the prepared particles revealed compatibility among ingredients as illustrated from the Fourier-transformed infrared and differential scanning calorimetry of the drug, PVA, physical mixture and the optimized formulation (Ahmed 2016). X-ray powder diffraction demonstrated crystalline state transformation from the crystalline state in the pure drug powder into the amorphous form in the optimized freeze-dried drug particles (Ahmed 2016).

### FSD calibration curve in plasma and tissue homogenate using NIR spectroscopy

Recently, NIR spectroscopy technique has gained a great interest and a wide range of application within the pharmaceutical industry for testing of raw material, product quality control and process monitoring. This great interest is perhaps a direct consequence of the technique major advantages over other currently used analytical techniques (Siddiqui et al., 2017). Following analysis of the plasma and tissue homogenates samples spiked with FSD using NIR and calculation of the drug concentration from the spectra; selectivity, linearity, precision and accuracy of the method were calculated.

The characteristic FSD finger print region was detected between 9921 and 9924 cm^−1^. Selectivity of the method was confirmed in which no potential interference at the characteristic drug frequency region with endogenous components was detected. The calculated *R*^2^ value where found to be 0.9566. Intra and interday precision and accuracy were also confirmed. The relative standard deviation was found to be within 15% while, the accuracy was calculated and found in the range 85–115%. The limit of detection (LOQ) and limit of quantification (LOD) were found to be 1 and 5 ng/ml, respectively.

### Pharmacokinetics and tissue distribution of FSD formulations

**Figure 1.**
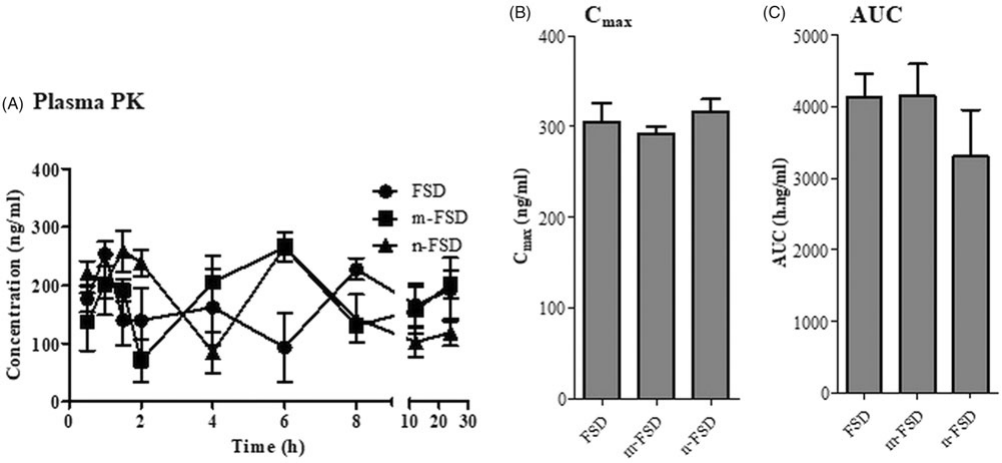
Plasma pharmacokinetics of FSD and its formulations. Different formulations of FSD (c-FSD, m-FSD and n-FSD) were administered to rats (0.4 mg/ml), blood samples were taken from retro-orbital plexus at different time points and plasma concentrations of FSD (A), maximum FSD concentration (B) and plasma overall exposure to FSD (C) were plotted. Data are expressed as mean ± SEM; *n* = 3; (*) indicates significantly different from corresponding control at *p* < .05.

**Figure 2.**
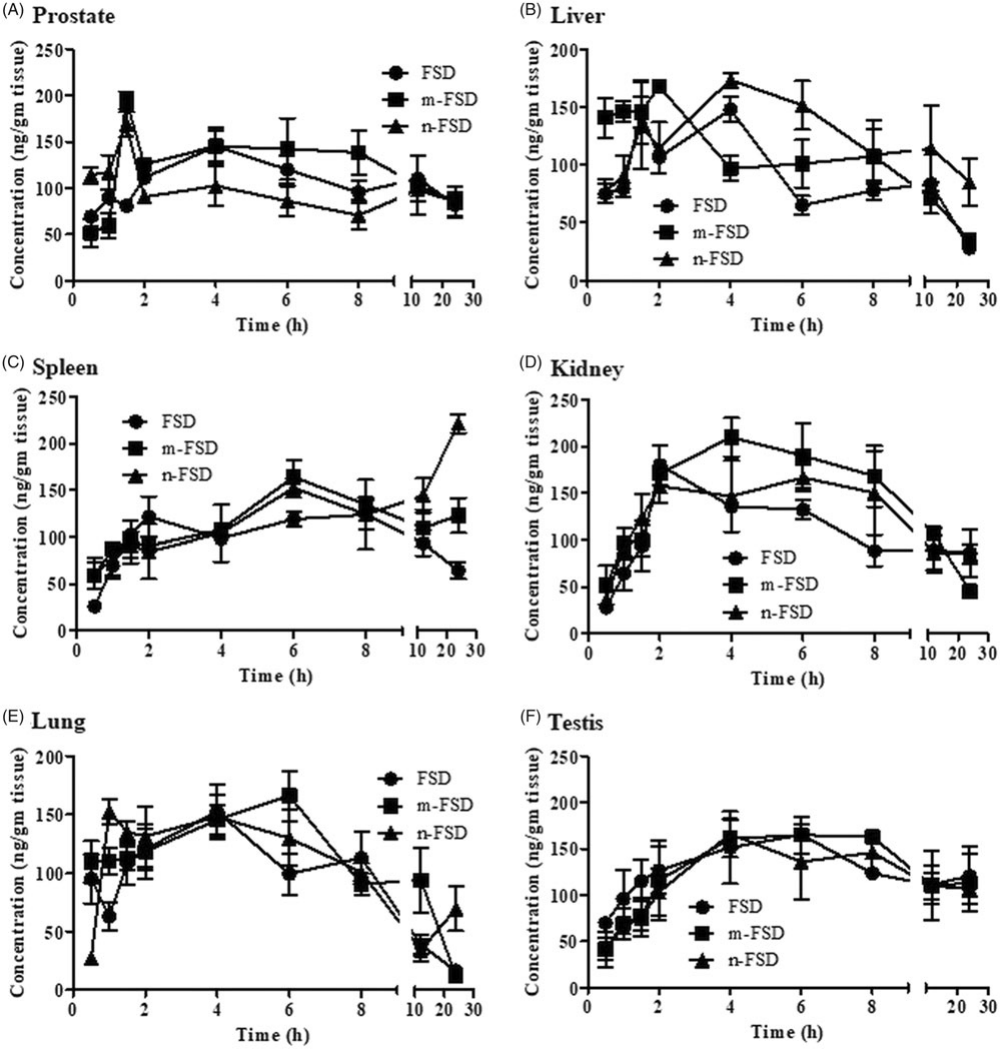
Tissue distribution of FSD and its formulations. Different formulations of FSD (c-FSD, m-FSD and n-FSD) were administered to rats (0.4 mg/ml), tissue samples such as prostate (A), liver (B), spleen (C), kidney (D), lung (E) and testis (F) were surgically extracted immediately after euthanasia and concentrations of FSD within the selected organs were plotted against time. Data are expressed as mean ± SEM; *n* = 3; (*) indicates significantly different from corresponding control at *p* < .05.

**Figure 3.**
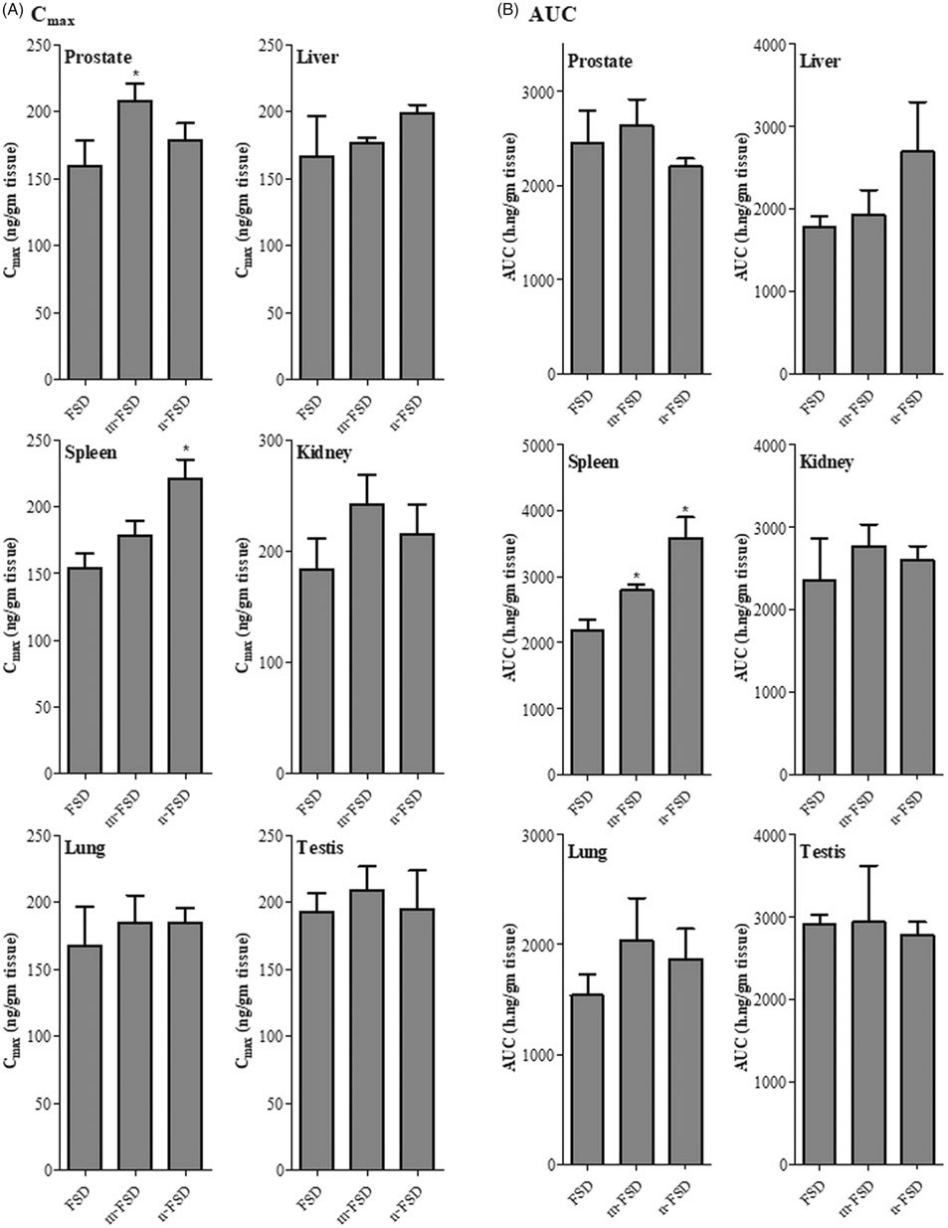
Tissue exposure parameters of FSD and its formulations. Different formulations of FSD (c-FSD, m-FSD and n-FSD) were administered to rats (0.4 mg/ml), tissue samples of prostate, liver, Spleen, Kidney, lung and testis were surgically extracted immediately after euthanasia and maximum concentrations (C_max_) of FSD (A) and total tissue exposure (AUC) to FSD were plotted. Data are expressed as mean ± SEM; *n* = 3; (*) indicates significantly different from corresponding control at *p* < .05.

After single oral administration of FSD (formulated and non-formulated) to normal rats, central compartment pharmacokinetics (plasma pharmacokinetics) and tissue distribution into target treatment tissue (prostate), nearby sex organ (testis), major reticuloendothelial/excretion organs (spleen, liver and kidney) and nonspecific random tissue (lung) were evaluated after different time points (Figures 2 and 3). Maximum plasma FSD concentration was achieved within 2 ± 0.7 h, 5.3 ± 0.5 h and 3.0 ± 1.1 h for c-FSD, m-FSD and n-FSD, respectively (Figure 1(a)). However, comparable C_max_ for c-FSD, m-FSD and n-FSD were observed (300.7 ± 14.6, 291.6 ± 6.4 and 317.1 ± 9.3 ng/ml, respectively) (Figure 1(b)). Similarly, comparable AUC’s for c-FSD, m-FSD and n-FSD were also observed (3.9 ± 0.2, 4.2 ± 0.3 and 3.3 ± 0.5 µg.h/ml, respectively); which are indicative of similar overall systemic exposure to FSD (Figure 1(c)). Maximum FSD concentration was achieved within prostate tissue after 6 ± 1.6 h and 5.2 ± 2.7 h for c-FSD and m-FSD, respectively. Interestingly, n-FSD managed to deposit FSD within prostate tissue as early as after 1.3 ± 0.2 h. However, comparable C_max_ (159.6 ± 13.5, 207.6 ± 9.3 and 178.9 ± 8.7 ng/g tissue) (Figure 3(a)) and AUC’s (2.5 ± 0.2, 2.6 ± 0.2 and 2.2 ± 0.1 µg h/g tissue) were noticed for c-FSD, m-FSD and n-FSD, respectively; which are indicative of similar overall prostate tissue exposure to FSD (Figure 2(a)). In previous studies, dihydrotestosterone (DHT) concentration within prostate tissue was taken to indirectly assess FSD effect rather than actual intra-prostatic FSD concentration (Wurzel et al., 2007).

Similarly, liver tissues showed comparable exposure parameters after treatment with c-FSD, m-FSD and n-FSD. C_max_’s were 166.3 ± 21.3, 176 ± 2.9 and 198.3 ± 4.8 ng/g tissue; T_max_’s were 3.2 ± 0.6, 1.3 ± 0.3 h and 4.5 ± 1.3 h (Figure 3(b)); and AUC’s were 1.8 ± 0.1, 1.9 ± 0.2 and 2.7 ± 0.4 µg h/g tissue for c-FSD, m-FSD and n-FSD, respectively (Figure 2(b)). Intra-hepatic FSD metabolism is crucial for its elimination, and relatively delayed hepatic T_max_ for n-FSD might improve its circulation time and prolong its activity (Ishii et al., 1994).

With respect to spleen tissue, c-FSD and m-FSD showed non-significantly different C_max_’s of 154.1 ± 7.6 and 177.7 ± 8.1 ng/g tissue, at T_max_’s of 5.3 ± 1.2 h and 4.5 ± 1.1 h, respectively. Only n-FSD showed higher C_max_ of 221.1 ± 10 ng/mg tissue at 22 ± 0.8 h (T_max_) (Figure 3(c)). This was significantly reflected as higher overall exposure of spleen tissue to FSD after m-FSD treatment (AUC = 2.8 ± 0.06 µg h/g tissue) and even higher after n-FSD treatment (AUC = 3.6 ± 0.2 µg h/g tissue) compared to c-FSD treatment (AUC = 2.1 ± 0.1 µg h/g tissue) (Figure 2(c)). Oral administration eliminates the influence of any reticuloendothelial system to the clearance of formulated drugs; as they must be dissolved to be absorbed (Zhang et al., 2008). Herein, FSD possess positive and beneficial influence to spleen-dependent erythropoietin secretion and activity (Barcelo et al., 1999).

Kidney is the major excretory organ for many drugs; intra-nephritic FSD concentration either reflects excretion or accumulation and nephropathy (Haque et al., 2017; Merkely et al., 2017). Fortunately, kidney tissues showed comparable exposure parameters after treatment with c-FSD, m-FSD and n-FSD. C_max_’s were 183.2 ± 19.9, 241.4 ± 19.4 and 214.8 ± 19.1 ng/g tissue (Figure 3(d)); T_max_’s were 3.3 ± 0.9, 5.2 ± 1.4 and 1.3 ± 0.1 h; and AUC’s were 2.4 ± 0.4, 2.8 ± 0.2 and 2.6 ± 0.1 µg h/g tissue for c-FSD, m-FSD and n-FSD, respectively (Figure 2(d)). FSD is not reported to undergo excessive renal clearance and kidney tissue does not possess FSD target enzyme and is not expected to be affected by FSD concentration (Steiner, 1996). Yet, renal tissue distribution of FSD might not be of high impact on either kidney or FSD itself.

Prostate and other male sex organs are sharing the same blood supply; drugs intended to target prostate such as FSD might additionally accumulate in other structurally adjacent or anatomically linked sex organ. It was reported that FSD might induce masculine-related side effects such as, erectile dysfunction (Zhang et al., 2012). Testis and testicular tissues are very sensitive to many drugs and to finastreride itself (Kolasa-Wolosiuk et al., 2015; Soni et al., 2017). Fortunately, FSD formulation did not influence its testicular maximum concentration (C_max_) or the overall testis exposure (AUC). C_max_’s were 192.8 ± 10.0, 208.7 ± 12.6 and 194.7 ± 20.4 ng/g tissue (Figure 3(f)); and AUC’s were 2.9 ± 0.1, 2.9 ± 0.5 and 2.8 ± 0.1 µg h/g tissue for c-FSD, m-FSD and n-FSD, respectively (Figure 2(f)).

Tissue distribution of FSD within lung was assessed as a representative of nonselective tissue distribution. To the best of our knowledge, no reports for the FSD accumulation within lung tissue (Steiner, 1996). Similarly, FSD formulation did not influence its maximum tissue concentration (C_max_) or the overall tissue exposure (AUC). C_max_’s were 167.0 ± 20.1, 184.1 ± 14.4 and 184.7 ± 7.9 ng/g tissue (Figure 3(e)); and AUC’s were 1.5 ± 0.1, 2.0 ± 0.3 and 1.9 ± 0.2 µg h/g tissue for c-FSD, m-FSD and n-FSD, respectively (Figure 2(e)).

### Cellular permeation of FSD formulations

**Figure 4.**
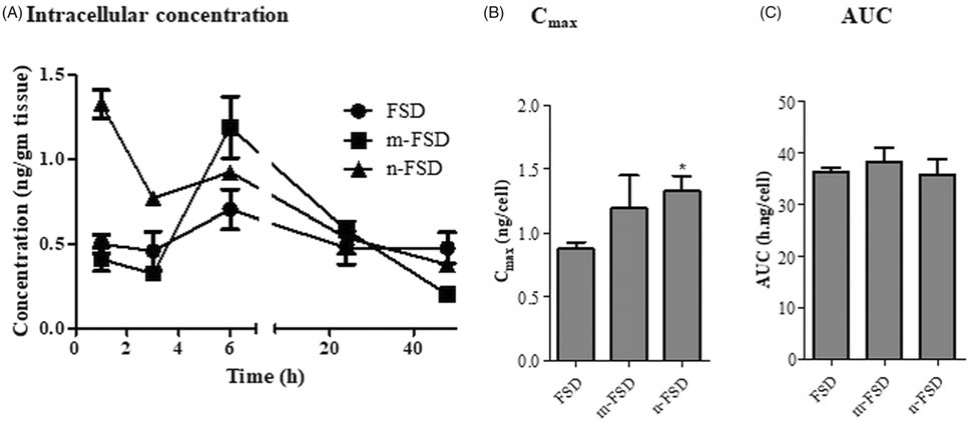
Cellular permeation of FSD and its formulations. Normal prostate epithelial cells (BPH cells) were exposed to 10 µg/ml different formulations of FSD (c-FSD, m-FSD and n-FSD), intracellular concentration of FSD were assessed after different time points (A), intracellular C_max_ (B) and intracellular AUC (C) were plotted. Data are expressed as mean ± SEM; *n* = 3; (*) indicates significantly different from corresponding control at *p* < .05.

FSD is known to exert its action via inhibiting the intracellular enzyme (type II 5α-reductase). Yet, FSD intracellular permeation is crucial for its activity. Herein, we assessed the cellular permeation of FSD and its different formulation (m-FSD and n-FSD) within normal prostate epithelial cells (PBH-1). Only, n-FSD significantly increased the intracellular FSD C_max_ and at earlier T_max_ (1.3 ± 0.1 ng/cell and 0.5 ± 0.01 h, respectively) compared to 0.9 ± 0.07 ng/cell and 4.2 ± 1.3 h for unformulated FSD. Unfortunately, m-FSD did not influence the intracellular Cmax of FSD nor its T_max_ (1.2 ± 0.2 ng/cell and 5.1 ± 0.1 h, respectively) (Figure 4). It is well-recognized that permeation assessment for any proposed 5α-reductase inhibitor is crucial rather than only assessing cell free enzyme inhibition (Mathur et al., 2004). Cellular permeation mechanisms are diverse such as, passive transport, active transport, endocytosis and phagocytosis. For the vast majority of particulate formulations (such as m-FSD and n-FSD), phagocytosis is expected to the be the predominating route of permeation (Lam et al., 1993; Gratton et al., 2008).

## Conclusions

In conclusion, nano-sized formulation of FSD significantly improved its cellular permeation. However, no significant changes in systemic pharmacokinetics (AUC and C_max_) were noticed due to size reduction. On the other hand, intra-prostatic accumulation was better for m-FSD rather than n-FSD and c-FSD; which could be explained by the higher reticuloendothelial uptake for n-FSD by the spleen.
